# Age of First Breeding Interacts with Pre- and Post-Recruitment Experience in Shaping Breeding Phenology in a Long-Lived Gull

**DOI:** 10.1371/journal.pone.0082093

**Published:** 2013-12-04

**Authors:** Davy S. Bosman, Harry J. P. Vercruijsse, Eric W. M. Stienen, Magda Vincx, Luc Lens

**Affiliations:** 1 Terrestrial Ecology Unit, Department of Biology, Ghent University, Ghent, Belgium; 2 Research Institute for Nature and Forest, Brussels, Belgium; 3 Marine Biology Section, Department of Biology, Ghent University, Ghent, Belgium; Université de Sherbrooke, Canada

## Abstract

Individual variation in timing of breeding is a key factor affecting adaptation to environmental change, yet our basic understanding of the causes of such individual variation is incomplete. This study tests several hypotheses for age-related variation in the breeding timing of Lesser Black-backed Gulls, based on a 13 year longitudinal data set that allows to decouple effects of age, previous prospecting behavior, and years of breeding experience on arrival timing at the colony. At the population level, age of first breeding was significantly associated with timing of arrival and survival, i.e. individuals tended to arrive later if they postponed their recruitment, and individuals recruiting at the age of 4 years survived best. However, up to 81% of the temporal variation in arrival dates was explained by within-individual effects. When excluding the pre-recruitment period, the effect of increasing age on advanced arrival was estimated at 11 days, with prior breeding experience accounting for a 7 days advance and postponed breeding for a 4 days delay. Overall, results of this study show that delayed age of first breeding can serve to advance arrival date (days after December 1^st^) in successive breeding seasons throughout an individual’s lifetime, in large part due to the benefits of learning or experience gained during prospecting. However, prospecting and the associated delay in breeding also bear a survival cost, possibly because prospectors have been forced to delay through competition with breeders. More generally, results of this study set the stage for exploring integrated temporal shifts in phenology, resource allocation and reproductive strategies during individual lifecycles of long-lived migratory species.

## Introduction

Individual variation in reproductive performance is an omnipresent feature of the demography of natural populations [1]. As evolutionary change is generated by variation in individual performance, understanding the processes driving this variation is fundamental to gain insight into individual behavioral strategies, life-history evolution and population dynamics [2,3]. The influence of age on reproductive performance is widely recognized and patterns of improved performance in early life, an asymptote at middle age, and evidence of senescence in older individuals, have been described in many long-lived mammals and birds, e.g., [4-11].

In migratory species, a key life-history trait associated with reproductive performance is the timing of individual appearance on the breeding grounds [12]. In seasonally reproducing animals, the timing of reproduction is very important and subject to intense selection pressures, because parents are expected to time their reproduction such that maximum offspring food requirements coincide with maximum food availability to ensure offspring survival [13]. Hence, a timely arrival on the breeding grounds constitutes an important precondition of successful reproduction, because it affects the length of the period available for breeding and can buffer yearly variation in peak food availability that determines the optimal onset of breeding. In migratory birds, early arrival at the breeding grounds has also been shown to result in priority access to high quality territories and nesting sites, e.g., [12]. Moreover, the evidence that early nesting confers advantage in terms of reproductive success (e.g. highest survival of nestlings) is overwhelming (reviewed in [14]). Although early arrival may thus improve reproductive success, it may also lead to considerable survival costs, e.g. as a result of inclement weather [15]. It is generally believed that the timing of arrival on the breeding grounds has important fitness consequences and hence is considered to constitute a key fitness parameter, e.g., [16,17]. 

While age is commonly regarded as a key parameter in explaining temporal variation in arrival at the breeding grounds, with older individuals usually arriving before younger ones, e.g., [16,18,19], age-related variation in timing of arrival has almost exclusively been studied from a between-individual (population) perspective. Hence, little is known about within-individual patterns of age-related variation. Two major hypotheses, not mutually exclusive, have been suggested to explain improvements in reproductive performance with age at the individual level and age-related competence and resource allocation [20,21]. The *experience* or *improvement of competence* hypothesis [1,8,21] states that reproductive performance may improve with age as a result of increasing experience, but only if experience enhances competency. This makes intuitive sense, as long-lived animals may take several years to acquire the skills necessary to forage efficiently, to obtain and defend good-quality territories or mates, which may have consequences for their arrival timing. This hypothesis is sometimes also referred to as the ‘constraint hypothesis’, suggesting that the lack of accumulated skill constrains the reproductive performance of young individuals [21]. By contrast, the *trade-off* or *effort* hypothesis argues that age-related improvement in reproductive performance is driven by increasing an individual’s level of reproductive investment owing to changes in reproductive costs or residual reproductive value, and implies a trade-off between current reproductive effort and future survival and fecundity [1,8,21]. This hypothesis is sometimes also referred to as the ‘restraint hypothesis’, suggesting that reproductive effort should be withheld early in life, if it has a disproportionately negative effect on future survival and breeding probabilities [21].

Another important factor underlying an improvement in reproductive performance with age might be the age of first breeding [8]. In long-lived migrants, the pre-reproductive period varies in length among individuals. Intraspecific variation in recruitment age may reflect quality of individuals [12] with lower-quality individuals experiencing higher costs and higher mortality associated with early arrival on the breeding grounds [22]. For some species there is indeed evidence that high-quality individuals are the first to arrive ([23] and references herein). The *delayed breeding* or *recruitment* hypothesis [24,25] proposes that these high-quality individuals delay their first breeding and recruit into the population at a later age (e.g. awaiting the opportunity to select a high-quality breeding territory) because early acquisition of a low-quality territory may lead to life-long low reproductive success due to high breeding site fidelity [26]. This progressive appearance of early arrivers into the breeding population may result in an advancement of arrival timing with age at the population level, even though no change is apparent at the individual level.

Delayed maturity is also often associated with the occurrence of a high proportion of pre-breeders at the breeding grounds, also known as prospectors [12,27]. During prospecting, individuals are believed to gather knowledge about potential breeding partners, territories, foraging sites and food supply, which may allow for a better integration into the breeding population afterwards. In support of this, several studies showed direct or indirect fitness benefits from prospecting behaviour prior to the first breeding attempt, e.g., [28-33], also in relation to arrival timing. For instance, in Common tern (*Sterna hirundo*), former prospectors arrived significantly earlier in the breeding colony during their reproductive life [29].

Finally, the *selection* hypothesis holds that if individuals that tend to arrive later also tend to die at a younger age, they will be underrepresented in the older age classes. As a consequence of these unobserved differences in survival abilities across individuals (commonly called frailty; [34]), arrival timing will advance with age at the population level, but again no change must have taken place at the individual level.

Here we study mechanisms underlying temporal variation in timing of arrival among and within individuals of the iteroparous Lesser Black-backed Gull (*Larus fuscus*), based on the analysis of a longitudinal dataset of phenological records from prospecting and breeding individuals in a coastal colony in NW Belgium spanning 13 annual cycles. Earlier studies in this colony showed that the yearly per capita number of fledglings is inversely related to laying date (Bosman unpublished data), while immature birds return later to the breeding grounds than breeding adults [35]. By taking advantage of the fact that age and age of first breeding are known from a large number of individually-marked birds, we here aim to investigate and disentangle effects of age, previous prospecting behavior, and years of breeding experience on arrival timing at the breeding colony. Within the framework of the non-mutually exclusive hypotheses outlined above, we address the following questions: (i) To what extent does timing of arrival constitute a dynamic trait (i.e. resulting from changes within individuals over time) or a fixed, consistent individual trait (i.e. resulting from phenotypic variation among individuals)? To disentangle within- from between-individual sources of variation in arrival dates and quantify their relative contribution, we used linear mixed-effects models. Modeling individuals and their longitudinal measurements as nested random effects thereby allows us to split total variance into a between-individual (σ^2^_*u*_) and a within-individual (σ^2^_*e*_; residual variance) component [36]; (ii) What is the role of experience gained from earlier prospecting or breeding?; (iii) Is there a correlation between variation in age of first breeding (α) or age of last breeding (ω) and age-related variation in timing of arrival?; (iv) To what extent is survival probability affected by early arrival and/or prospecting behaviour at early age?

## Materials and Methods

### Ethics statement

This study was conducted under research permits EC2012-052 and EC2013-027 from the Animal Ethics Committee of Ghent University. Permission to capture and handle birds was granted by the Flemish Nature and Forest Agency (Brussels). Upon capture, all necessary steps were taken to minimize animal suffering. No birds were kept in captivity, sacrificed or injured, and no tissue or blood sampling was conducted for this study. Immediately after ringing, all individuals were released in perfect body condition. Access to the study area was provided by the Port Authorities of Zeebrugge and by the associated bird ringing group housed at the Royal Belgian Institute of Natural Sciences (Brussels).

### Study species, field procedures and data collection

*Larus fuscus* of the subspecies *graellsii* are long-distance migrants between their main wintering areas in Iberia and western North Africa and their breeding grounds in NW Europe [37], where they breed in mixed colonies with Herring Gull (*Larus argentatus*). Our study colony is located in the outer port of Zeebrugge (Belgium, 51°21’N, 03°11’E) and hosts up to ± 4500 pairs of *Larus fuscus* annually. In this study, we analyzed phenology data collected between 1999 and 2012, a timeframe spanning 13 annual cycles (database managed by the Research Institute of Nature and Forest, Belgium). Only established breeding birds that were individually marked as nestlings and therefore of known age, were considered for analysis. On a total ringing effort of 1664 nestlings up to 2009, 310 individuals of different birth cohorts for which arrival data were collected, survived to breeding age and established themselves in our study colony. Breeding adults were sexed on the basis of direct size comparison of paired individuals, complemented by observations of copulation and courtship behavior at the breeding colony. The repeatability of sex assessment of individuals recorded during subsequent breeding seasons equaled 100%. The transition from migration to breeding was defined as the earliest sighting in the colony each year, based on meticulous observations conducted from morning till evening every second day between mid-winter till the start of egg-laying. For each sexually mature *Larus fuscus* of known age, individual arrival dates were subsequently calculated as the number of days since 1 December of the previous year (the nominal starting point in our population); see also [38]. Up to 2012, we obtained comprehensive data on the breeding experience of 211 individuals, calculated as the number of reproductive years accumulated before the current breeding season. Each year, highly experienced observers recorded the activity of all individually-ringed birds throughout the breeding season, taking GPS coordinates of each nest during early nest-building and marking all nests with an individually coded stick. Throughout the study, individuals showed a high degree of nest site fidelity which facilitated their early detection in the colony and allowed ample time to search for new recruits. Given the high level of nest site fidelity, the high search effort by multiple experienced observers from nest-building till fledging, and the high and constant resighting probability during the study period (see Results), we believe that our breeding data were both highly accurate and complete. Realized age of first breeding (α), defined as the age when an individual was first recorded breeding, ranged between 3 to 7 years (µ = 4.09 years ± 0.05 years). To be conservative, we assumed that only individuals that were not resighted in the breeding colony during at least three consecutive years and that had not been resighted during migration, at their wintering grounds, or in other well-studied neighboring colonies in the southern part of the North Sea: Nord, Pas-de-Calais (France); Zeeland, Noord-Brabant, Zuid- and Noord-Holland (the Netherlands); Suffolk (United Kingdom); Schleswig-Holstein (Germany), were dead. In very rare occasions birds were observed to loose uniquely-coded rings, however this was actively countered by targeted re-ringing campaigns during each breeding season. Applying these criteria, realized age of last breeding (ω) was known for 66 individuals, of which six were actually reported dead. Data on prospecting behavior before recruitment, inferred from the earliest sighting in number of days for each year (see above), were available for 150 breeding individuals.

### Statistical analysis and hypothesis testing

Within- and between-individual variation in timing of arrival was analyzed by linear mixed-effects models (LMM; [39]) in SAS 9.3 (SAS Institute Inc., Cary, NC, USA). The analyzed datasets and the SAS-syntax may be found in Appendixes S1 and S2, respectively. In all models, random intercepts and slopes were included to account for variance among individuals and for non-independency of repeated measures from the same individual, respectively. By modeling individuals and their longitudinal measurements as random effects, we accounted for between-individual variation in strength of relationships (i.e. slope of response) with other variables (e.g. advanced arrival with age) [40]. In addition, individual identity was modeled as a repeated effect with autoregressive covariance structure to control for covariance between pairs of observations, given that repeated measures closer in time are likely to be more strongly correlated [10,39]. We used the Kenward-Roger denominator degrees of freedom method to correct for downward bias in standard error estimates in the covariance matrix. As year and age are likely correlated in studies of individually-marked animals unless they continue over decades, we included year as a random factor in all models. To test for age-related variation in timing of arrival, age was modeled both as linear and quadratic (age*age) effect with arrival date as dependent variable. As age effects are predicted to be more pronounced when non-breeding individuals are included [20], we additionally modeled pre-recruitment (prospecting) arrival dates of subsequent breeders to compare age effect sizes on timing of arrival between analyses that either included or excluded these data. As factor sex did not significantly explain variation in timing of arrival in any of the models including survival analysis (data not shown), data from males (n = 179), females (n = 53) and unsexed individuals (n= 78) were subsequently pooled.

Age-related variation in arrival timing was first examined at the population level. We analyzed all arrival dates, i.e. including those related to prospecting behavior (n = 1364), while in a second model we only included arrivals directly related to reproduction (n = 1134). However, because 12 of these pooled individuals were mates (i.e. belonging to six breeding pairs), they were nested (Table 1, upper panels) to avoid pseudoreplication at the level of the breeding pair. 

**Table 1:** Linear mixed models testing the linear and quadratic effects of age, age of first breeding (α) and age of last reproduction (ω) on arrival date including and excluding pre-recruitment arrivals.

	breeders (**incl**. prospecting years)					breeders (**excl**. prospecting years)				
	Estimate	SE	d.f.	F	*p*	Estimate	SE	d.f.	F	*p*
Intercept	195.06	3.61	—	—	—	172.10	5.99	—	—	—
Age	-14.78	1.12	497	175.45	**<0.0001**	-8.66	1.67	478	26.97	**<0.0001**
Age²	0.77	0.08	351	97.49	**<0.0001**	0.41	0.11	350	14.76	**0.0001**
Random variance (Year)	-0.23	0.37	—	—	—	-0.64	0.43	—	—	—
Individual (nested in pair) variance (intercept)	83.19	48.77	—	—	—	118.58	76.92	—	—	—
Individual (nested in pair) variance (slope)	0.94	1.36	—	—	—	0.92	1.76	—	—	—
Autogressive covariance	0.08	0.05	—	—	—	0.08	0.06	—	—	—
Residual variance	558.41	28.57	—	—	—	540.01	32.81	—	—	—
Intercept	201.20	10.10	—	—	—	164.47	13.73	—	—	—
Age	-23.64	2.12	120	124.94	**<0.0001**	-13.25	3.92	83.2	11.44	**0.001**
Age²	1.46	0.18	62	64.73	**<0.0001**	0.74	0.28	50.1	6.83	**0.01**
α	5.08	2.21	63.4	5.28	**0.02**	5.72	2.83	58.8	4.07	**0.04**
ω	-1.22	0.87	53.9	1.96	0.17	-1.25	1.12	69.7	1.24	0.27
Random variance (Year)	0.02	1.51	—	—	—	-0.31	2.69	—	—	—
Individual variance (intercept)	23.24	51.31	—	—	—	78.40	106.73	—	—	—
Individual variance (slope)	3.74	2.69	—	—	—	2.41	3.76	—	—	—
Autogressive covariance	0.05	0.09	—	—	—	0.12	0.12	—	—	—
Residual variance	342.86	32.96	—	—	—	297.28	42.88	—	—	—

To test predictions stemming from the *selection* and *recruitment* hypotheses, we built a LMM including parameters age of first breeding (α) and age of last reproduction (ω) as explanatory variables (pre-recruitment arrivals included: n= 352 dates; pre-recruitment arrivals excluded: n= 254 dates; Table 1, lower panels) and a third model with age of first breeding (α) only (n=1048 and n= 818, respectively; Table 2, upper panels). As curvilinear relationships with fitness performances were documented in vertebrates before [10,41], we initially tested for quadratic relationships with α and ω. However, as these effects were not significant at the 5% probability level (data not shown), they were not withheld in our final models. If early individuals survive better, we expect a negative relationship between arrival date and ω in accordance with the selection hypothesis. If early individuals delay recruitment into the breeding population until a later age, we expect a negative relationship between arrival date and α as predicted by the recruitment hypothesis. A positive relationship between arrival date and α, in contrast, would be consistent with a more advanced arrival with accumulating breeding experience in individuals of similar age [20].

**Table 2:** Linear mixed models testing the linear and quadratic effects of breeding experience (EXP) and age of first breeding (α) on arrival date including and excluding pre-recruitment arrivals.

	breeders (**incl**. prospecting years)					breeders (**excl**. prospecting years)				
	Estimate	SE	d.f.	F	*p*	Estimate	SE	d.f.	F	*p*
Intercept	181.75	5.86	—	—	—	159.79	6.70	—	—	—
Age	-18.37	1.09	379	285.56	**<0.0001**	-11.12	1.61	320	47.44	**<0.0001**
Age²	1.01	0.08	268	165.66	**<0.0001**	0.58	0.10	302	31.68	**<0.0001**
α	4.79	1.27	201	14.13	**0.0002**	3.72	1.44	191	6.65	**0.01**
Random variance (Year)	-0.32	0.29	—	—	—	-0.24	0.38	—	—	—
Individual variance (intercept)	99.19	37.73	—	—	—	103.10	60.99	—	—	—
Individual variance (slope)	-0.62	1.15	—	—	—	-0.37	1.50	—	—	—
Autoregressive covariance	0.15	0.05	—	—	—	0.15	0.06	—	—	—
Residual variance	414.02	23.96	—	—	—	331.34	24.72	—	—	—
residual variance	148.66	5.25	—	—	—	140.59	5.40	—	—	—
EXP	-11.68	1.03	361	128.47	**<0.0001**	-7.18	0.94	396	57.92	**<0.0001**
EXP²	1.05	0.14	89.6	56.15	**<0.0001**	0.70	0.12	86	31.69	**<0.0001**
α	0.27	1.24	201	0.05	0.83	-0.15	1.29	182	0.01	0.91
Random variance (Year)	-0.48	0.32	—	—	—	-0.12	0.38	—	—	—
Individual variance (intercept)	58.80	33.43	—	—	—	81.43	38.23	—	—	—
Individual variance (slope)	4.95	2.68	—	—	—	3.89	2.59	—	—	—
Autoregressive covariance	0.25	0.05	—	—	—	0.18	0.07	—	—	—
Residual variance	532.40	32.78	—	—	—	337.32	26.07	—	—	—

To test whether sexual maturation and experience gained from earlier prospecting or breeding explain average within-individual variation in timing of arrival as predicted by the *experience* hypothesis, we first performed two additional LLMs modeling breeding experience (EXP) while controlling for the effect of α (n= 1048 and n= 818 arrival dates when including and excluding pre-recruitment arrivals, respectively; Table 2, lower panels). In a subsequent LMM (Table 3, left panel), we tested whether or not prospection before recruitment (0 or 1) was significantly related to individual timing of arrival during reproduction (n= 818 arrival dates). In a final LMM (Table 3, right panel), we constricted individual life-histories of breeding individuals to a three-level fixed factor (pre-recruitment/prospecting, first-time breeding or experienced breeding) as a measure of degree of sexual maturation, and tested whether the timing of arrival spanning the entire lifetime significantly varied among these life-stages (n= 1048 arrival dates). In these last two models, we initially controlled for α, but removed this factor after backward selection (results not shown). Because age and breeding experience are highly correlated, we substituted linear and quadratic covariates of age by breeding experience (EXP and EXP²) when testing the constraint hypothesis, cf., [10]. Because of the occurrence of prospecting behavior before recruitment and/or instances of intermittent breeding after recruitment, age was modeled as a categorical variable in the repeated statement to specify the order of each observation within each individual. First-order interactions with EXP and EXP² were not significant in all analyses and were sequentially removed in a backward selection procedure.

**Table 3:** Linear mixed models testing the linear and quadratic effects of breeding experience (EXP) and the effects of prospecting behavior excluding pre-recruitment arrivals and maturation including pre-recruitment arrivals on arrival dates.

	breeders (excl. prospecting years)						breeders (incl. prospecting years)				
	Estimate	SE	d.f.	F	p		Estimate	SE	d.f.	F	p
Intercept	145.07	2.09	—	—	—	Intercept	161.87	1.59	—	—	—
EXP	-7.08	0.93	392	58.56	**<0.0001**	EXP	-5.13	1.63	295	9.92	**0.001**
EXP²	0.70	0.12	91	32.63	**<0.0001**	EXP²	0.46	0.19	63.9	6.18	**0.02**
Prospect	-7.33	2.30	194	10.15	**0.002**	Maturation	—	—	743	65.86	**<0.0001**
						Prospects	0	—	—	—	—
						First-time breeders	-20.60	1.98	—	—	—
						Experienced	-25.63	3.03	—	—	—
Random variance (Year)	-0.10	0.35	—	—	—	Random variance (Year)	-0.27	0.31	—	—	—
Individual variance (intercept)	78.07	34.11	—	—	—	Individual variance (intercept)	89.85	33.11	—	—	—
Individual variance (slope)	3.90	2.56				Individual variance (slope)	4.15	2.50			
Autogressive covariance	0.14	0.06	—	—	—	Autogressive covariance	0.14	0.05	—	—	—
Residual variance	326.81	23.82	—	—	—	Residual variance	429.94	25.21	—	—	—

### Survival analysis

To test whether early investment in reproductive performance has a negative effect on survival as predicted by the *trade-off* hypothesis, we performed two survival analyses. The analyzed datasets may also be found in Appendix S1. In these analyses, we investigated the survival cost of early arrival during the year of first reproduction, rather than during the year of first arrival in the colony, because not all individuals prospected and different processes might drive the arrival timing of prospectors and first-time breeders. Additionally, we examined if the absence or presence of prospecting behavior affected survival chances. We also included age of first breeding and its quadratic effect, because stabilizing selection on recruitment age through differential survival has previously been found in gulls [42]. 

First, we performed a multi-state CMR-analysis in MARK 7.0 (Colorado State University, USA). Within the framework of this study, birds of known age were initially marked as immatures (STATE A) and matured when recruiting into the breeding population (STATE B). Hence, realized age of first breeding was defined as the maturation point. Next, we imposed a series of logical constraints based on a priori knowledge of the realized life-histories of the individuals under study here. Since only individuals that survived to maturity were included, the parameter for immature survival was fixed to S^i^ = 1. Next, variation in realized age of first breeding was known to be age-related (3 to 7 years old). Therefore, we modeled an age-dependent transition probability to maturity (Ψ^i→m^) with six age steps (≤2,3,4,5,6,≥7), without recruitment probability before the age of 3 (a_1_ = 0), and with a recruitment probability of 1 for individuals of age 7 years or older (a_6_ = 1; i.e. knowing that all individuals still alive at this age had recruited). Once an individual matured, it remains so and maturity hence constitutes an absorbing state (Ψ^m→i^ = 0). Since we primarily aimed to model variation in mature survival after recruitment (S^m^), immaturity was regarded an unobservable state with zero resighting probability (p^i^ = 0). Individual resighting histories were restricted to 198 individuals for which (i) initial release data as pullus (age=0), (ii) arrival dates in the first year of reproduction, and (iii) resightings as mature breeding bird within the colony in subsequent years after recruitment were available. The dataset also contained three individual-level covariates, i.e. the timing of arrival during the first year of reproduction (AIFY), the absence or presence of prospecting behavior during pre-recruitment (PROS = 0 or 1) and age of first breeding (α and its quadratic effect coded as power(α,2) in the design matrix). As few birds recruited at age 6 and 7 (n =9 individuals), we restricted α to three levels (3 years; 4 years; 5 years or older) in order to obtain reliable estimates of the mature survival parameters as a function of α (see Results). We used a starting model with time variation in mature survival and resighting probabilities, i.e. S^m^(t) p^m^ (t) Ψ^i→m^ (age). Being a reduced parameter general model, we applied a median-ĉ GOF test to assess the goodness-of-fit (GOF) of this starting model (see web-based manual to MARK, chapter 8). Subsequently, the results were corrected for slight over-dispersion of the data using the value of the GOF parameter ĉ = 1.67 ± 0.08 (lower bound =1.0, upper bound = 5.0, 10 design points with 100 replicates at each point). Next, a series of candidate models were fitted that differed in the extent to which mature survival and resighting rates were held constant (indicated with S^m^(.) and p^m^ (.) respectively) or whether S^m^ was considered to be a function of AIFY, PROS, α and/or α^2^. Model selection methods were based on Akaike Information Criterion (AIC; [43]) and candidate models were ranked by second-order AIC differences (Δ AIC_c_). Only models that deviated less than 2 AIC_c_ units from the most parsimonious model (ΔAIC_c_ = 0) were considered to have approximately equal weight in the data in accordance with model weights and evidence ratios presented by [44]. To be conservative, models that deviated >2 AIC_c_ units from the most parsimonious model were considered to be unsupported by our data (see web-based manual to MARK, chapter 5). The model averaging procedure was used to compute the average estimates for mature survival and resighting probabilities based on weighted AIC_c_-values for each model and thus accounts for model uncertainty in these estimates.

Second, we built a backward stepwise Cox proportional hazards Model (Proc PHREG) in SAS 9.3 (SAS Institute Inc., Cary, NC, USA) to relate the risk of death after recruitment (i.e. hazard) to (i) the timing of arrival during the first year of reproduction (AIFY), (ii) the absence or presence of prospecting behavior during pre-recruitment (PROS = 0 or 1), and (iii) the age of first breeding (α) and its quadratic effect (n = 198 individuals, ties = breslow). TIME*STATUS was modeled as the response variable, where TIME refers to the follow-up time (in years) after recruitment and STATUS is the event indicator with value 1 for death time and value 0 for censored time. When performing the Cox regression, α was modeled as a continuous variable.

## Results

### Age-specific phenology, selection and recruitment hypotheses

**Figure 1:**
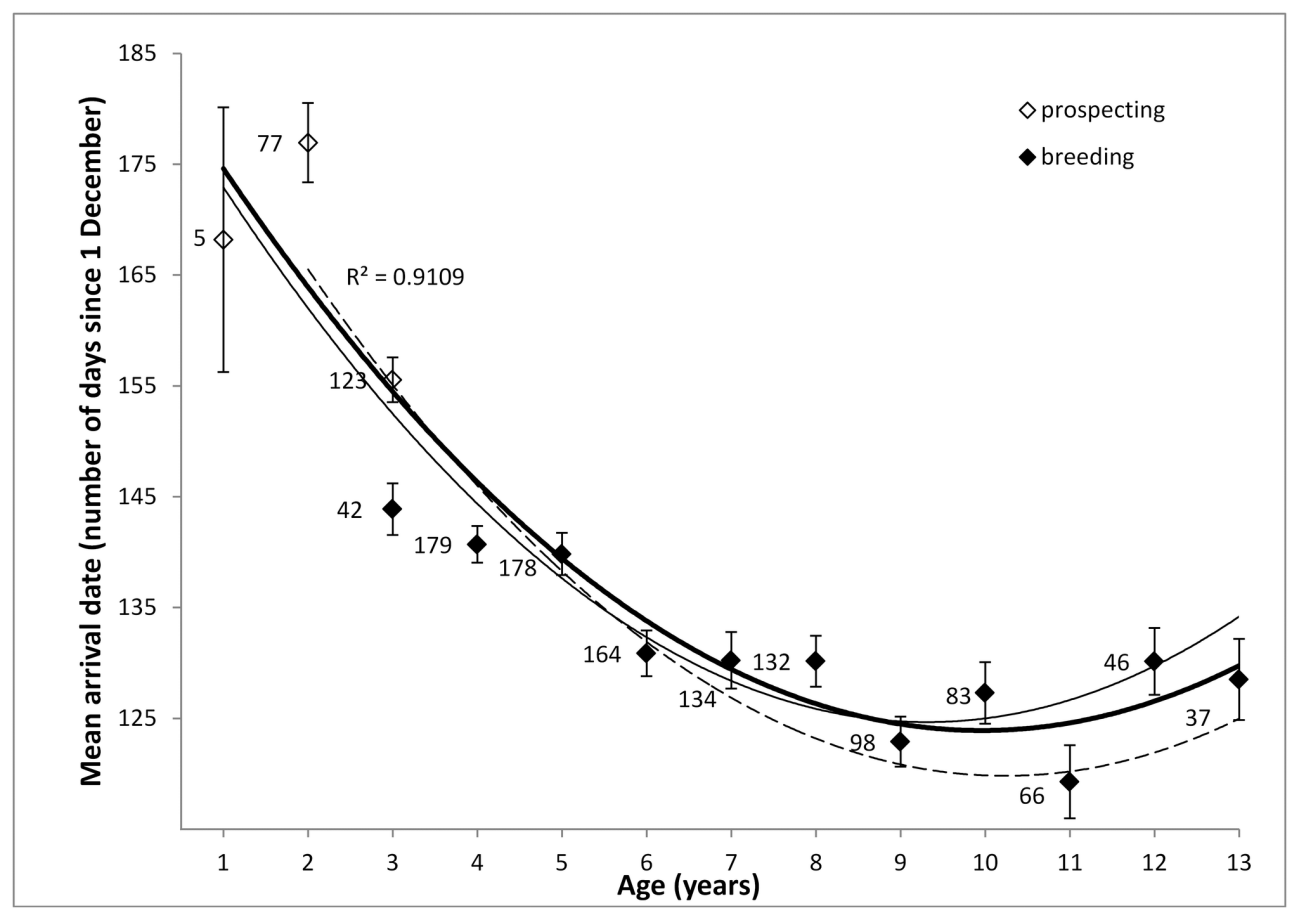
Progressive advancement in mean arrival date (± SE) with age at the population level. For 310 *Larus fuscus* from a Belgian breeding colony spanning 13 annual cycles (solid bold trendline). Numbers refer to sample sizes. Male (n = 179) and female (n = 53) trendlines are depicted by solid and dashed thin lines respectively. Pre- and post-recruitment arrival data are depicted by open and filled symbols, respectively.

Timing of arrival at the breeding colony showed linear and quadratic relationships with age (Tables 1 and 2, Figure 1), and the positive quadratic effect indicated that the advance of arrival date with age decreased when individuals grew older. Contrary to the selection hypothesis, the age when individuals disappeared (ω) did not significantly explain variation in timing of arrival (Table 1). The effect of age of first breeding (α) on arrival date was significant, however, the relationship was positive whereas a negative relationship was predicted by the recruitment hypothesis. When controlling for the significant effect of α, individual identity accounted for and 19% and 24% of the total variance (calculated as the sum of the individual variance component and the residual variance) in arrival dates when including or excluding pre-recruitment arrival dates, respectively (Table 2 upper panel). These percentages reflect a common measure of repeatability (R) and hence quantify the constancy of phenotypes, while 1-R can be considered a measure of phenotypic plasticity, cf., [45]. Hence, within-individual effects explained 81% and 76% of the total variation in arrival dates when including or excluding pre-recruitment arrival dates, respectively. Overall, effect sizes of relationships with age were stronger when pre-recruitment arrival dates were included (Tables 1 and 2). 

### Experience hypothesis

**Figure 2:**
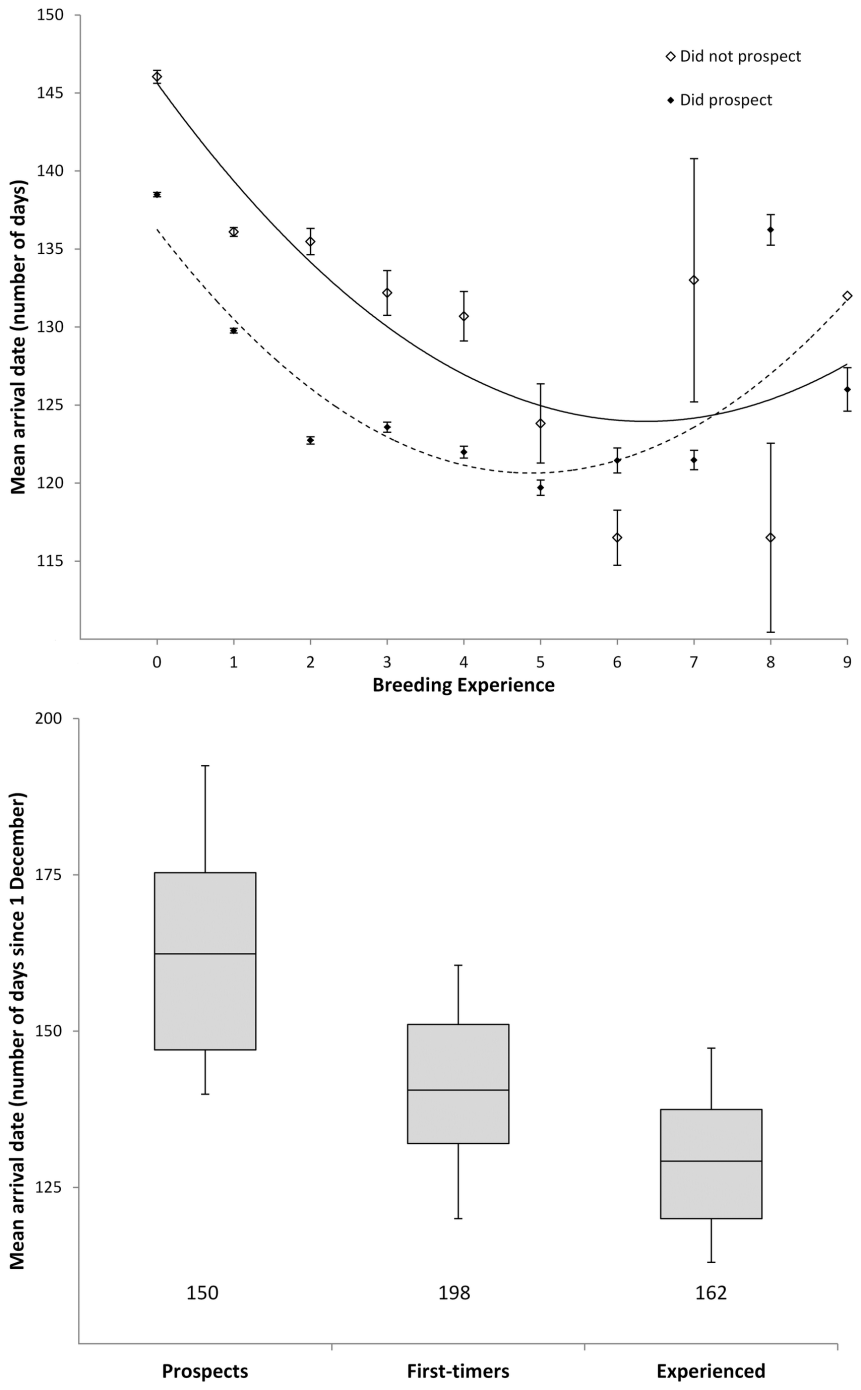
Relationship between breeding experience and mean arrival date (± SE). 211 *Larus fuscus* from a Belgian breeding colony spanning 10 breeding cycles were grouped by whether they prospected (n = 150) or not (n = 61) and depicted by filled and open symbols, and dashed and solid trendlines, respectively (upper panel). The lower panel shows the same individuals grouped by successive stages of sexual maturity. The box and whiskers plots represent the distribution of arrival dates for each group, with means (solid lines), 10% and 90% (whiskers), and 25% and 75% (box) quartiles Numbers refer to sample.

Individuals that delayed their first breeding tended to arrive consistently later at the colony throughout their lifetime, but nevertheless advanced their arrival with increasing age. This pattern, and the fact that the effect of α on arrival date was strongly reduced when accounting for breeding experience (Table 2), is consistent with the hypothesis that breeding experience may underlie the positive relationship between α and timing of arrival (Table 2). Individuals indeed showed linear and quadratic advances in arrival date with accumulating levels of EXP (Table 2 and 3). Additionally, sexual maturation and experience drawn from prospecting behaviour both explained variation in arrival dates while accounting for EXP, i.e. resulting in a further advancement of timing of arrival with age (Table 3, Figure 2). 

### Trade-off hypothesis

Both survival analyses yielded highly comparable results (probabilities ± SE). Of the candidate set of CMR models, the one with a constant probability of mature survival (S^m^(.) = 0.91 ± 0.02; weighted average) and a constant resighting probability (p^m^(.) = 0.97 ± 0.01; weighted average) fitted best to our data (Table 4). All models that related variation in mature survival to timing of arrival in the first year of reproduction (AIFY), deviated considerably from this most parsimonious model (2 < ΔQAIC_c_ > 7; Table 4) and were therefore considered unsupported by our data. In contrast, four models that related variation in mature survival to the effect of age of first breeding (α), prospecting behaviour (PROS) or both, had ΔQAIC_c_ values of less than two (Table 4) and were therefore considered equally informative as the most parsimonious model. Prospecting behaviour during pre-recruitment was inversely related to mature survival (S^m^ = 2.53 – 0.35(PROS)) with a slightly lower survival probability for prospectors (S^m^ = 0.89 ± 0.02) compared to non-prospectors (S^m^ = 0.93 ± 0.03). As adult survival was highest in individuals that recruited at the age of 4 (S^m^ = 0.92 ± 0.02) and lower in both early (age of 3: S^m^ = 0.86 ± 0.04) and late (age of 5 or older: S^m^ = 0.88 ± 0.03) recruiters, our survival data support the notion of normalizing selection acting on age of first breeding (S^m^ = -7.27 + 4.81(α) - 0.59(α^2^)). Likewise, mature survival was inversely related to the occurrence of prospecting behaviour before recruitment in a Cox proportional hazards model (β = 1.34 ±0.54, χ^2^ = 6.18, p = 0.01; -2 log likelihood = 480.393, Global Score χ^2^ = 8.91, p = 0.03), with a fourfold increase in mortality risk after recruitment (Hazard ratio = 3.83). While mature survival was also significantly related to both α (β = -2.83 ± 1.17, χ^2^ = 5.76, p = 0.02) and α^2^ (β = 0.33 ± 0.54, χ^2^ = 5.98, p = 0.02) with minimal mortality risk when recruiting at the age of 4 (data not shown), no significant relationship was apparent between AIFY and mortality risk following recruitment into the breeding population (χ^2^ = 2.10, p = 0.99).

**Table 4:** Results of multi-state CMR model selection on survival and recapture probabilities in *Larus fuscus*.

Model	QAIC_c_	Δ QAIC_c_	QAIC_c_ Weight	Model Likelihood	NP	Qdeviance
S^m^(.) p^m^ (.) Ψ^i→m^ (age)	641.7845	0.0000	0.2531	1.0000	11	619.4782
S^m^(.) **α α^2^** p^m^ (.) Ψ^i→m^ (age)	642.7719	0.9874	0.1545	0.6104	13	616.3487
S^m^(.) **PROS** p^m^ (.) Ψ^i→m^ (age)	643.2420	1.4575	0.1221	0.4825	12	618.8797
S^m^(.) **PROS α α^2^** p^m^ (.) Ψ^i→m^ (age)	643.3955	1.6110	0.1131	0.4469	14	614.9066
S^m^(.) **α** p^m^ (.) Ψ^i→m^ (age)	643.7775	1.9930	0.0934	0.3692	12	619.4151
S^m^(.) **AIFY α α^2^** p^m^ (.) Ψ^i→m^ (age)	644.4558	2.6713	0.0666	0.2630	14	615.9668
S^m^(.) **AIFY PROS** p^m^ (.) Ψ^i→m^ (age)	645.2409	3.4564	0.0450	0.1776	13	618.8176
S^m^(.) **PROS α** p^m^ (.) Ψ^i→m^ (age)	645.2560	3.4715	0.0446	0.1763	13	618.8327
S^m^(.) **AIFY PROS α α^2^** p^m^ (.) Ψ^i→m^ (age)	645.3464	3.5619	0.0427	0.1685	15	614.7869
S^m^(.) **AIFY α** p^m^ (.) Ψ^i→m^ (age)	645.6632	3.8787	0.0364	0.1438	13	619.2399
S^m^(.) **AIFY PROS α** p^m^ (.) Ψ^i→m^ (age)	647.2519	5.4674	0.0165	0.0650	14	618.7629
S^m^(.) **AIFY** p^m^ (.) Ψ^i→m^ (age)	647.9150	6.1305	0.0118	0.0467	12	623.5526
S^m^(.) p^m^ (t) Ψ^i→m^ (age)	655.5974	13.8129	0.0003	0.0010	26	601.9398
S^m^(t) p^m^ (.) Ψ^i→m^ (age)	664.7807	22.9962	0.0000	0.0000	26	611.1231
S^m^(t) p^m^ (t) Ψ^i→m^ (age)	677.5672	35.7827	0.0000	0.0000	41	591.4278

Goodness-of-fit of the starting model was assessed by a median-ĉ GOF test and the relative fit of alternative models was assessed by Akaike’s Information Criterion. The number of parameters in each model is indicated by NP. The following parameters were fixed in all models : S^i^ = 1, p^i^ = 0, Ψ^m→i^ = 0 and for Ψ^i→m^ (age): a_1_ = 0 and a_6_ = 1 (see Methods for details and rationale).

## Discussion

In our study population of *Larus fuscus*, age of an individual has a significant effect on its phenology, with older age classes arriving progressively earlier at the breeding grounds (Figure 1). After statistical deconstruction of the within- and between-individual processes underpinning this pattern, we found support for age-related variation in arrival timing at both the population and individual levels. In particular, variation in Larus fuscus phenology was related to recruitment age and experience gained from earlier prospecting or breeding.

### Arrival timing and recruitment age

At the population level, age of first breeding (α) was significantly associated with both survival and arrival timing in *Larus fuscus*. Individuals tended to arrive significantly later if they postponed their recruitment, which countered the overall trend of advanced arrival with increasing age and opposes the predictions of the recruitment hypothesis. As we suspect that early recruits might possess inherent reproductive, competitive and cognitive abilities that could allow them to arrive earlier than individuals that delay recruitment, cf., [12,34], recruitment at young age might be an indicator of higher quality in *Larus fuscus*. Our study further showed that individuals recruiting at the age of 4 years had the highest survival probability. Reduced survival chances for later recruits (α > 4 years old) suggest that these birds may be of lower phenotypic quality and therefore less able to cope with costs associated with early arrival [15]. Such conclusion supports those of other studies that showed lower reproductive performance in more strongly delayed breeders, e.g., [7,10,46,47]. Nevertheless, recruiting at the earliest possible age may not be the best strategy either. According to life-history theory, individuals should begin to reproduce at an age when the net benefits are greater than delaying reproduction [48]. Earlier recruits (α < 4 years old) may consistently arrive too early throughout their lifetime and as a consequence pay the elevated cost of reduced survival probability. Hence, progressive appearance of late arriving *Larus fuscus* at the population level may jointly reflect the disappearance of phenotypes that consistently arrive too early and the appearance of low-quality phenotypes that consistently arrive later than average recruits (α = 4 years old) of the same age during subsequent years. This supports the idea that timing of arrival in *L. fuscus* is at least to some degree a consistent individual trait, and evidence for (partial) individual consistency in timing of arrival and migration was earlier shown in other species of birds, e.g., Common Tern (*Sterna hirundo*), [12]; Pied Avocet (*Recurvirostra avosetta*), [16]; Snow Goose (*Anser caerulescens*), [49]; and Marsh Harrier (*Circus aeruginosus*) [50], and fishes; Roach (*Rutilus rutilus*), [51].

### Arrival timing and breeding experience

Despite indications for consistent variation in timing of arrival among individuals belonging to different recruitment age groups, up to 81% of the temporal variation in arrival dates in our population was explained by within-individual effects. Similarly, 87% of the variation in reproductive performance in a longitudinal study on *Sterna hirundo* was explained by within-individual changes [9]. Although it is tempting to argue that this is primarily related to progressive gain in breeding experience with age (experience hypothesis; table 2), the confounded nature of both variables renders it difficult, if not impossible, to study their independent effects on arrival date. Nevertheless, over the entire reproductive lifetime of *Larus fuscus* (i.e. when excluding the pre-recruitment period), the effect of growing age on advanced arrival was estimated at 11 days, with prior breeding experience accounting for a 7 days advance and postponed breeding for a 4 days delay (Table 2, right panel). This suggests that accumulating breeding experience may be at least one important factor explaining advanced arrival over an individual’s reproductive lifetime. Likewise, the role of breeding experience was considered strong in the long-lived Greater Flamingo (*Phoenicopterus roseus*) where breeding propensity was largely determined by increasing levels of experience [52]. When including the pre-recruitment years in our study, however, arrival advanced with 18 and 12 days in relation to age and breeding experience respectively, while the delay related to postponed breeding remained comparable (Table 2, left panel). This discrepancy suggests that additional factors may trigger variation in timing of arrival during immature (pre-recruitment) life-stages. 

### Arrival timing and previous prospecting behavior

One additional trigger of variation in timing of arrival during pre-recruitment may be that non-recruited individuals, and to lesser extent first-time breeders, are still subject to maturation of (sexual) function, which may constrain their navigational skills, foraging or breeding abilities. While post-recruitment experience can improve reproductive performance through previous breeding opportunities (see above), pre-recruitment experience may help to achieve higher levels of reproductive performance through prospection. During prospection, immature and subadult birds may improve these skills and thereby facilitate a timely, stepwise transition from migration to breeding [12]. In support of this [53], showed a strong reduction in annual distribution range of *Larus fuscus* as maturing individuals progressively remained closer to the breeding grounds year-round. Furthermore, prospectors arrived on average seven days earlier at the breeding colony throughout the larger part of their reproductive life, compared to individuals that did not show such behaviour (Table 3; Figure 2, left panel). However, in *Larus fuscus*, prospecting behaviour also comes with a considerable survival cost, which may explain why half of the breeders that indulged in prospecting prior to recruitment, restricted this behaviour to a single season at the age of three years or older. Prospecting is presumably costly owing to higher competition for food resources around colonies, more abundant parasites and higher risk of aggressive encounters with conspecifics [28], while time and energy spent during prospection may also be traded off against other activities, such as foraging and resting [54].

### Selection and trade-off hypotheses

We did not find evidence that selective disappearance contributed to the observed population-level arrival trajectory. The absence of a significant effect of age of last reproduction (ω) on timing of arrival renders it unlikely that age-related variation in arrival dates resulted from a selective disappearance of late arrivers with increasing age, as predicted by the selection hypothesis. While 66 individuals with known or inferred realized age of last breeding may appear low in terms of sample size when testing for selective disappearance, our results are strongly in line with the weak evidence for differential survival commonly reported from other long-lived species in which low annual mortality (ca. 10% for *Larus fuscus*; [55], this study) may only cause minor changes in the distribution of early and late individuals among age classes [20], but see [7]. Additionally, survival probabilities did not increase with more advanced arrival early in reproductive life which further disproved the selection hypothesis, cf., [7].

Likewise, and contrary to the predictions from the trade-off hypothesis, we did not find direct evidence for the existence of a trade-off between current reproductive effort and future survival. Specifically, more advanced arrival dates early in reproductive life did not reduce survival chances later on. Although lowered adult survival probabilities through survival costs related to prospecting behaviour and through very early recruitment when only 3 years old (see above)—both in turn related to a more advanced arrival timing—may indirectly affect an individual’s timing effort with increasing age.

Overall, our findings demonstrate that age, recruitment age and individual experience may interact in shaping patterns of variation in timing of arrival in *Larus fuscus*. The advancement of arrival date occurred primarily at the individual level and can be explained mainly by a gain in individual experience with increasing age, although we did not succeed in fully disentangling the roles of the latter two factors. As previously shown in Black-legged Kittiwake (*Rissa tridactyla*), delaying recruitment up to intermediate ages may be associated with fitness advantages that offset the costs of delayed maturity [56]. In *Larus fuscus*, individuals recruiting at the age of 4 balanced pre- and post-recruitment experience in an advantageous way, advanced their arrival timing with age accordingly and achieved the highest survival probability through stabilizing selection acting on age of first breeding.

While in many species, arrival dates at breeding sites significantly vary between sexes [19,57], an earlier study in the same *L. fuscus* colony showed highly synchronized arrival dates of male and female breeders [38]. We currently lack sufficient data to directly relate timing of arrival to individual laying dates. However, if present, such relationship might still be obscured by age-related variation in breeding and resource allocation strategies. For example, late individuals might make disproportional use of capital (rather than income) resources to advance their laying date in the face of a seasonal decline in reproductive success, e.g., Snow Goose (*Caerulescens caerulescens*) [58]. Hence, to assess to what extent age-specific strategies in timing of arrival affect lifetime reproductive success in long-lived migrant species, future studies need to explore integrated temporal shifts in phenology, resource allocation and reproductive strategies during individual lifecycles, cf., [9]. We thereby predict ageing individuals to shift along the income-capital allocation continuum, relative to parallel shifts in migratory and reproductive strategies.

In conclusion, this study has shown that a complex interplay between a fixed trait (age of first breeding) and the balancing of pre- and post-recruitment experience can shape a dynamic trait, i.e. age-related advancement in timing of arrival.

## Supporting Information

Appendix S1 **Analyzed datasets.**
(XLSX)

Appendix S2 **SAS-syntax for the linear mixed-effects models.**
(PDF)
