# Supplementary material for: Age of First Breeding Interacts with Pre- and Post-Recruitment Experience in Shaping Breeding Phenology in a Long-Lived Gull
Source: PLoS One. 2013 Dec 4;8(12):e82093. doi: 10.1371/journal.pone.0082093 (PMC3852959; doi:10.1371/journal.pone.0082093)
Supplement: Appendix S2 — SAS-syntax for the linear mixed-effects models. (PDF) [file pone.0082093.s002.pdf]

SAS syntax Table 1; upper two panels.

```
proc mixed covtest nobound;
class code pair ageclass;
model arrival = age age*age/solution ddfm=kr;
random intercept age age*age year/subject=code(pair);
repeated ageclass /subject=code(pair) type=ar(1);
run;
```

SAS syntax Table 1; lower two panels.

```
proc mixed covtest nobound;
class code ageclass;
model arrival = age age*age α ω/solution ddfm=kr;
random intercept age age*age year /subject=code;
repeated ageclass /subject=code type=ar(1);
run;
```

SAS syntax Table 2; upper two panels.

```
proc mixed covtest nobound;
class code ageclass;
model arrival = age age*age α /solution ddfm=kr;
random intercept age age*age year /subject=code;
repeated ageclass /subject=code type=ar(1);
run;
```

SAS syntax Table 2; upper two panels.

```
proc mixed covtest nobound;
class code ageclass;
model arrival = exp exp*exp α /solution ddfm=kr;
random intercept exp exp*exp year/subject=code;
repeated ageclass/subject=code type=ar(1);
run;
```

SAS syntax Table 3; left panel.

```
proc mixed covtest nobound;
class code ageclass;
model arrival = exp exp*exp prospect/solution ddfm=kr;
random intercept exp exp*exp year /subject=code;
repeated ageclass/subject=code type=ar(1);
run;
```

SAS syntax Table 3; right panel.

```
proc mixed covtest nobound;
class code maturation ageclass;
model arrival = maturation exp exp*exp /solution ddfm=kr;
random intercept exp exp*exp year /subject=code;
repeated ageclass/subject=code type=ar(1);
run;
```
